# Author Correction: The consequences of tetraploidy on *Caenorhabditis elegans* physiology and sensitivity to chemotherapeutics

**DOI:** 10.1038/s41598-024-66715-5

**Published:** 2024-07-09

**Authors:** Kelly R. Misare, Elizabeth A. Ampolini, Hyland C. Gonzalez, Kaitlan A. Sullivan, Xin Li, Camille Miller, Bintou Sosseh, Jaclyn B. Dunne, Christina Voelkel-Johnson, Kacy L. Gordon, Jessica H. Hartman

**Affiliations:** 1 Department of Biochemistry and Molecular Biology, College of Medicine, Medical University of South Carolina, Charleston, SC 29425 USA; 2 Department of Biology, College of Arts and Sciences, University of North Carolina, Chapel Hill, NC 27599 USA; 3 Department of Microbiology and Immunology, College of Medicine, Medical University of South Carolina, Charleston, SC 29425 USA

Correction to: *Scientific Reports* 10.1038/s41598-023-45225-w, published online 23 October 2023

The Acknowledgments section in the original version of this Article was incorrect.

“The authors would like to thank Dr. Mara Schvarzstein (Brooklyn College-CUNY, New York, NY) for her kind assistance in establishing tetraploid lines and Jason Pierce in the Lipidomics Core for his assistance with preparing samples for lipidomics. This work was supported in part by the American Cancer Society Institutional Research Grant #IRG-19-137-20, from the American Cancer Society, the Hollings Cancer Center at the Medical University of South Carolina, the National Institutes of Health R00-ES029552 (JHH), R35-GM150843 (JHH), R35-GM147704 (KLG), T32-GM132055 (EAA and JBD), R25-GM113278 (HCG) and the Lipidomics Shared Resource, Hollings Cancer Center, Medical University of South Carolina (P30 CA138313 and P30 GM103339).”

now reads:

“The authors would like to thank Dr. Mara Schvarzstein (Brooklyn College-CUNY, New York, NY) for her kind assistance in establishing tetraploid lines and Jason Pierce in the Lipidomics Core for his assistance with preparing samples for lipidomics. This work was supported in part by the American Cancer Society Institutional Research Grant #IRG-19-137-20, from the American Cancer Society, the Hollings Cancer Center at the Medical University of South Carolina, the National Institutes of Health R00-ES029552 (JHH), R35-GM147704 (KLG), T32-GM132055 (EAA and JBD), R25-GM113278 (HCG) and the Lipidomics Shared Resource, Hollings Cancer Center, Medical University of South Carolina (P30 CA138313 and P30 GM103339).”

The original Article has been corrected.

